# ICAM-1-LFA-1 Dependent CD8+ T-Lymphocyte Aggregation in Tumor Tissue Prevents Recirculation to Draining Lymph Nodes

**DOI:** 10.3389/fimmu.2018.02084

**Published:** 2018-09-12

**Authors:** Alba Yanguas, Saray Garasa, Álvaro Teijeira, Cristina Aubá, Ignacio Melero, Ana Rouzaut

**Affiliations:** ^1^Department of Biochemistry and Genetics, University of Navarra, Pamplona, Spain; ^2^Program of Immunology and Immunotherapy, Center for Applied Medical Research (CIMA), Pamplona, Spain; ^3^Centro de Investigación Biomédica en Red Cáncer, CIBERONC, Madrid Spain; ^4^Department of Plastic and Reconstructive Surgery, Clínica Universidad de Navarra, Pamplona, Spain; ^5^Immunology and Immunotherapy Unit, Clínica Universidad de Navarra, Pamplona, Spain

**Keywords:** draining lymph nodes, T-lymphocyte retention, homotypic cell-adhesion, ICAM-1, immune response, cancer immunotherapy

## Abstract

The quantity of T-lymphocytes reaching the draining lymph nodes from tumors is likely important to mount effective distant responses and for the establishment of long term systemic memory. Looking into mechanisms behind lymphocyte egress, we directed our attention to leukocyte adhesion mechanisms inside tumors. Here we demonstrate that activated T-cells form intra-tumor aggregates in a LFA-1-ICAM-1-dependent fashion in mouse models of melanoma and breast cancer. We also provide evidence of the presence of T-cell clusters in primary human melanoma. Disruption of LFA-1-ICAM-1 interactions, and thereby T-cell clustering, enhances the arrival of activated CD8+ T-cells to tumor draining lymph nodes in both transplanted and spontaneous cancer models. Interestingly, upon ICAM-1 blockade, the expression of the chemotactic receptor CCR7 augments in tumor infiltrating lymphocytes and in *in-vitro* de-clustered T cells, as well as their ability to transmigrate across lymphatic endothelial cells. We propose that ICAM-1-mediated homotypic T-lymphocyte aggregation may serve as a tumor-mediated immune retention mechanism entrapping activated CD8+ T cells in the tumor microenvironment. Modulation of T-cell adhesion may be of use to improve the transit of activated lymphocytes toward the lymph nodes and their subsequent recirculation.

## Introduction

Failure of T cell infiltration into tumors or in their recirculation from the tumor niche to the draining lymph nodes (LNs) are some of the factors behind lack of therapeutic effects in patients treated with immunotherapy. To develop competent cytotoxic functions, CD8+ T lymphocytes must be efficiently primed by professional antigen presenting cells in the LNs (1). For this purpose, naïve CD8+ T cells constantly traffic through secondary lymphoid organs where they systematically scan the surface of dendritic cells searching for specific antigens (2, 3). In fact, T lymphocytes are the major leukocyte cell type in steady-state afferent lymph (4).

Once they have been activated in the LNs, T cells travel to tumor affected tissues following chemokine gradients to eradicate malignancy. Accordingly, patients with a pre-existing immune infiltrate are better responders to check-point blockade immunotherapy (5). For cold tumors, improved T-lymphocyte entrance can be achieved by induction of chemokines such as CXCL10, CCL2, or CCL5, that recruit T cells (6), by blood vessel normalization or by depletion of suppressive immune cells that hamper the entrance of tumor infiltrating lymphocytes (TILs) (7).

However, T lymphocyte function goes beyond primary tumor killing. Recent evidences show how antigen-experienced T cells emigrate from tissues to secondary lymphoid organs following chemotactic cues such as CCL21 or S1P (8–10). Memory CD4+ T lymphocytes and at much lower amounts CD8+ memory T cells leave inflamed peripheral tissues through the lymphatic vessels (LVs) (11). Although the molecular routes that regulate T-cell egress from inflamed tissues have been extensively studied it is far from clear whether the same mechanisms are at work in leukocyte egress from tumors (12). Interestingly, recent reports by Torcellan and co-workers demonstrated by *in vivo* photolabeling of subcutaneous tumors, that tumor-egressing T-cells constitute an heterogeneous population that includes relatively high numbers of CD4+ and CD8+ T lymphocytes with effector phenotypes and moderate amounts of IL-17 producing CD4-CD8- double negative T lymphocytes (13). At this moment, whether the lymph nodes constitute a transitory location for effector lymphocytes traveling to distant metastases or a place for further reactivation of memory T cells is an issue of research.

Different soluble and stroma-bound signals are responsible of lymphocyte retention or egress from inflamed tissues. For example, in the small intestine epithelium, brain and skin epidermis, stromal TFGβ reduces the expression of T-bet by resident memory T cells leading to activation of the integrin αE (CD103) locus and T cell residence in the tissue by adhesion to its ligand E-cadherin. In contrast, lamina propria memory T cells that do not express CD103 depend on macrophages and antigen-derived stimuli for lymphocyte retention (14). Lymphocyte retention can also be accomplished by avoidance of exit cues present in the stroma. Among them, inhibition of the egress receptors sphingosine-1-phosphate receptor 1 (S1P1) (15) or CCR7 (16).

In addition, tumors co-opt the adhesive mechanisms used in inflamed tissues to regulate lymphocyte activation and positioning within their stroma. In this sense, T-cell integrins and their cognate ligands expressed on target cells, mainly lymphocyte-function-associated antigen-1 (LFA-1)/intercellular adhesion molecule-1 (ICAM-1) and CD103/E-cadherin play a relevant role in the interactions between cytotoxic T lymphocytes and tumor cells (17, 18). For instance, it has been reported in breast tumor models how the reactivation of effector T cells mostly depends on their binding to cognate antigen presented by tumor infiltrating CD103 expressing dendritic cells (19). In addition, chemokines secreted by the inflamed stroma contribute to homotypic and heterotypic intratumoral T cell adhesion, for example regulating the avidity/affinity of key integrins such as LFA-1 (20).

In this study, we explored the role played by the LFA-1 ligand ICAM-1 in T cell retention in the tumor milieu. In a previous work, we studied the intervention of the integrin ligands ICAM-1 or VCAM in leukocyte transmigration across the lymphatic endothelium under inflammation (21). Moreover, the role of ICAM-1/LFA-1 pairs in T cell crawling on initial lymphatics has been recently addressed (22). However, nobody has investigated yet the role played by ICAM-1 in tumor infiltrating lymphocytes' exit from tumor.

To address this issue, we blocked ICAM-1 in mice that next received intratumoral injections of activated T-lymphocytes. To our surprise, we observed significant increases in the transit of CD8+ T cells to the lymph nodes in LFA-1/ICAM-1 blocked animals. The same increments were observed in a spontaneous model of breast cancer. In all these cases, ICAM-1 blockade led to *in vitro* and *in vivo* decrease of T-cell aggregates or clusters, with a parallel increment in oriented cell migration and transmigration across monolayers of lymphatic endothelial cells.

Therefore, since LFA-1/ICAM-1 T cell aggregation seems to limit T-cell recirculation, transient local blockade of these functions offers opportunity to attain systemic bio-distribution of tumor-reactive T-lymphocytes. Although, lack of data makes debatable whether T-cell egress from tumors is a meaningful phenomenon in cancer immunology (23), our results suggest that modulation of LFA-1/ICAM-1 to implement T-lymphocyte egress from malignant tissue is a possibility.

## Materials and methods

### Mice and cell lines

C57BL/6 female mice (6–7 weeks old) were obtained from Harlan Laboratories and kept in our institutional animal facility following ethical guidelines. OT1, OT1 CD45.1, and Her2/Neu transgenic mice were bred in our laboratory. All procedures were carried out in compliance with European Union and University of Navarra (Institutional Animal Care and Use Committee Protocol n° 168-12) relevant guidelines for the use of laboratory animals.

Immortalized mouse lymphatic endothelial cells (IMLEC) were cultured at 33°C on collagen (Corning Life Sciences, Corning, NY) and fibronectin (Millipore, Billerica, MA) coated dishes (both 10 μg/ml). Murine interferon-γ (IFNγ; 10 U/ml, R&D Systems, Abingdon, UK) was added to induce the expression of the large T-antigen during the expansion period. IMLEC culture media was 40% DMEM, 40% F12-Ham, 20% FBS (all from Gibco, Carlsbad, CA), 56 μg/ml heparin (Sigma Aldrich, St. Louis, MO), 10 μg/ml endothelial cell mitogen (AbD Serotec, Düsseldorf, Germany) and antibiotic antimycotic solution (Life Technologies, Madrid, Spain). Lymphatic vessels identity was confirmed by staining with antibodies against CD31 (BD Bioscience, Madrid, Spain), podoplanin and LYVE-1 (both form Abcam, Cambridge, UK). Before each experiment IFNγ was removed from the cell growth media for at least 72 h.

B16-F10-OVA melanoma cells, which express the OVA257 epitope, and B16-F10-VEGFC cells (kindly donated by Dr. Melody Swartz, Lausanne, Switzerland), which express the vascular endothelial growth factor C, were cultured in complete media containing RPMI 1640 medium (Gibco) supplemented with 10% FBS, 100 U/ml penicillin/streptomycin (Gibco) and 5 × 10^−5^ M 2-mercaptoethanol (Gibco).

B16-F10-VEGFC-OVA cells were obtained by transfection using lipofectamine 2000 (Thermo Fisher Scientific, Waltham, MA). Twenty-four hours before transfection, 10^5^ B16-VEGFC cells were seeded in 6 well plates in 2 ml complete media to reach 70% confluence on the day of transfection. Cells were then transfected with a plasmid containing OVA, and 72 h after transfection, selection with geneticin 1 mg/ml was started and maintained for 1 week. Expression of OVA and VEGFC by stably transfected cells was checked by flow cytometry using FACSCanto (BD Bioscience).

### *In vitro* CD8+ T-lymphocyte activation

Naïve OT1 T cells (enriched from spleens of OT1 CD45.1 C57BL/6 mice) were activated by exposure to 100 ng/ml OVA. For clustering assays, 30 min prior to activation, 10 μg/ml anti-ICAM-1 mAb (clone YN1/1.7.4), anti-LFA-1 mAb (clone M17/4) or IgG2b kappa monoclonal isotype control (clone RTK4530; all from Biolegend, San Diego, CA) were added to the cell culture. Brightfield images of cell clusters were acquired 24 and 48 h after activation using an inverted microscope (Leica, Wetzlar, Germany). Image quantification was performed using ImageJ software. For de-clustering assays, OT1 lymphocytes which had been activated for 48 h were transferred to 96-well plates and treated with 10 μg/ml anti-ICAM-1, anti-LFA-1 or isotype control. Images were acquired 24 h after the addition of the mAbs and quantification was performed using ImageJ.

CD8+ T-lymphocytes from spleens of Balb/c mice were isolated using the CD8α T Cell Isolation kit (Miltenyi Biotec, Bergisch Gladbach, Germany) and following manufacturer's instructions. Cells were further activated with plate-bound anti-CD3ε (0.5 μg/ml; clone 145-2C11; Biolegend) and anti-CD28 (1 μg/ml; clone 37.51; Biolegend) in complete culture media. Two days later, CD8+ T cells were labeled with violet proliferation dye 450 (BD Bioscience) before being injected into the tumors.

### Migration assays

For transmigration assays, 4 × 10^4^ IMLEC were seeded on the top membrane of transwell Boyden chambers with a 5-μm pore size (Corning Life Sciences) pre-coated with type I rat tail collagen (Becton Dickinson, Franklin Lakes, NY). Cells were allowed to attach overnight at 37°C. One day later, TNF-α (Peprotech, London, UK) was added onto confluent endothelial cell monolayers at a final concentration of 100 ng/ml. Cells were maintained in culture for extra 12 and 1 h before the addition of lymphocytes, anti-ICAM-1 or anti-LFA-1 antibodies were added at a concentration of 10 μg/ml. Activated OT1 CD8+ T cells were first stained with deep red CellTracker (Thermo Fisher Scientific) and then exposed or not to blocking antibodies at the same concentration and time as IMLEC monolayers. Then, 10^5^ OT1 cells were added onto the IMLEC monolayer in the upper well of the transwell inserts. Cells were allowed to transmigrate for 12 h at 37°C. Migrated cells were collected from the lower compartment and quantified using a Neubauer chamber. Images of the remaining cells in the upper well of the Boyden chamber were acquired by confocal microscopy and quantified using Image J software.

To test lymphocyte 3D migration, OT1 T-lymphocytes were activated by 48 h-exposure to OVA in the presence of 10 μg/ml anti-ICAM-1 mAb or in the presence of a kappa monoclonal isotype control. Cells were stained with a CellTracker CMRA orange (Thermo Fisher Scientific) and embedded in 3D collagen matrices (PureCol EZ Gel solution, Sigma Aldrich). Lymphocyte movement toward CCL21 (SLC) gradients was recorded by *in vivo* confocal microscopy using LSM 800 with Airyscan (Zeiss, Jena, Germany). The cell directionality ratio is defined as the straight-line length between the starting point and the endpoint of the migration trajectory, divided by the total length of the trajectory. This ratio is equal to 1 for a straight cell trajectory and approaches 0 for a highly curved trajectory (24). It was analyzed using IMARIS software (Bitplane, Belfast, UK).

### Flow cytometry

OT1 T-lymphocytes activated for 48 h in the presence or absence of ICAM-1 blocking mAbs were stained with antibodies against the chemokine receptors CCR7 (clone 4B12; Biolegend). Single cell suspensions from tumors and lymph nodes were stained with antibodies against CD45.1 (clone A20, Biolegend) and counted using perfect count beads (Cytognos, Salamanca, Spain) as internal standards. Dead cells were excluded using the Zombie NIR Fixable viability kit (Biolegend). Cells were collected with FACSCanto II and cell acquisition data was analyzed using FlowJo software (Treestar, Ashland, OR).

### Confocal microscopy

For immunofluorescence-based analysis, samples were frozen, cut into 5 μm slices and stained overnight with 1:100 dilutions in TBS 1X of rabbit anti-mouse LYVE-1, rat anti-mouse CD8 (clone 53–6.7, Biolegend), PE anti-mouse CD45.1 (clone A20, Biolegend) or 488 anti-mouse CD11b (clone M1/70, Biolegend) antibodies. Isotype specific secondary antibodies conjugated to Alexa-Fluor 488, 594 or 647 (Life Technologies) were used as needed. Cell nuclei were stained with Hoechst 33258 (Life Technologies) and imaged on a LSM 800 confocal microscope (Zeiss) equipped with a 63X Apochromat (N/A = 1.4).

Image quantification was performed using ImageJ and LSM image analyzer software (Zeiss). For CD8+ T cell analysis, a region of interest (ROI) was determined manually. Mean fluorescence intensity (MFI) of CD8+ signal on each ROI was analyzed using ImageJ. CD8+ T cell clusters and CD8+ and CD11b co-staining was manually delimited and quantified using ImageJ. Aggregates of four or more cells were considered clusters.

### Immunohistochemistry

Formalin-fixed, paraffin-embedded melanoma tissue sections were obtained from remnant samples collected for clinically-indicated procedures after firmed consent. Endogenous peroxidase activity was quenched with hydrogen peroxide, and antigen retrieval was carried out in citrate buffer pH 6. Nonspecific binding was blocked using 5% normal goat serum in TBS for 30 min. Sections were incubated with anti-CD8a antibody (clone HPA037756, Atlas Antibodies, Bromma, Sweden) overnight at 4°C. Afterwards, samples were incubated with Envision polymer (Dako, Glostrup, Denmark) for 30 min at room temperature.

### *In vivo* studies

For *in vivo* migration experiments, C57BL/6 mice or Her2/Neu transgenic mice were used. C57BL/6 mice were injected subcutaneously with 5 × 10^5^ B16-VEGFC cells in both flanks. When tumors reached sizes of about 7 × 7 mm, tumors were inflamed by an intratumoral injection of 20 ng/ml of TNF-α. Two days later mice were treated intraperitoneally (i.p.) with anti-ICAM-1 (50 μg per mouse), anti–LFA-1 (100 μg per mouse) blocking mAbs or rat IgG2b isotype control (50 μg per mouse). Eight hours later C57BL/6 and Her2/Neu mice where adoptively transferred with 0.5 × 10^5^ activated OT1 CD45.1 cells or 0.3 × 10^5^ activated CD8+ T cells respectively. When indicated, mice were intravenously injected with 3 μg pertussis toxin (PTx) 4 h after mAb treatment (Sigma-Aldrich). Two days later, mice were humanly sacrificed and T-lymphocytes were recovered from tumors and draining lymph nodes. For this purpose, isolated tumors were incubated with Collagenase-D and DNase-I (Roche, Madrid, Spain) for 15 min at 37°C, followed by mechanical disaggregation and filtration in a 70-μm cell strainer (BD Bioscience). Tumor infiltrating lymphocytes were isolated from stromal cells through Percoll gradients. Lymph nodes were mechanically disaggregated and filtrated through a 70-μm cell strainer in order to obtain a single-cell suspension.

For *in vivo* migration experiments with naïve OT1 cells, C57BL/6 mice were injected subcutaneously with 5 × 10^5^ B16-VEGFC-OVA cells in both flanks. When tumors reached sizes of about 7 × 7 mm, tumors were inflamed with an intratumoral injection of 20 ng/ml of TNF-α. Forty-eight hours later, mice were adoptively transferred with intravenous injections of 5·10^6^ naïve OT1 lymphocytes. Two days later, mice were treated intraperitoneally with anti-ICAM-1 (50 μg per mouse) or rat IgG2b isotype control (50 μg per mouse) and daily intraperitoneal injections of 5 mg/kg FTY720 (fingolimod, Sigma). Two days later, mice were humanly sacrificed and T-lymphocytes were recovered from tumors and draining lymph nodes.

### Statistical analyses

Statistical analyses were performed using SPSS 17.0 software (SPSS, Chicago, IL, USA). Normal distribution was assured by use of the Kolmogorov–Smirnov test. If the distribution was normal, the significance was calculated with *t-*test or ANOVA (Shapiro–Wilk), if not, the significance was estimated by nonparametric tests such as Mann–Whitney *U*-test. In all cases, one (*), two (**), or three (***) asterisks indicate significances of *p* < 0.05, *p* < 0.01, and *p* < 0.001, respectively.

## Results

### ICAM-1 blockade increments the migration rates of intratumorally administered T-lymphocytes to the draining lymph nodes

**Figure 1:**
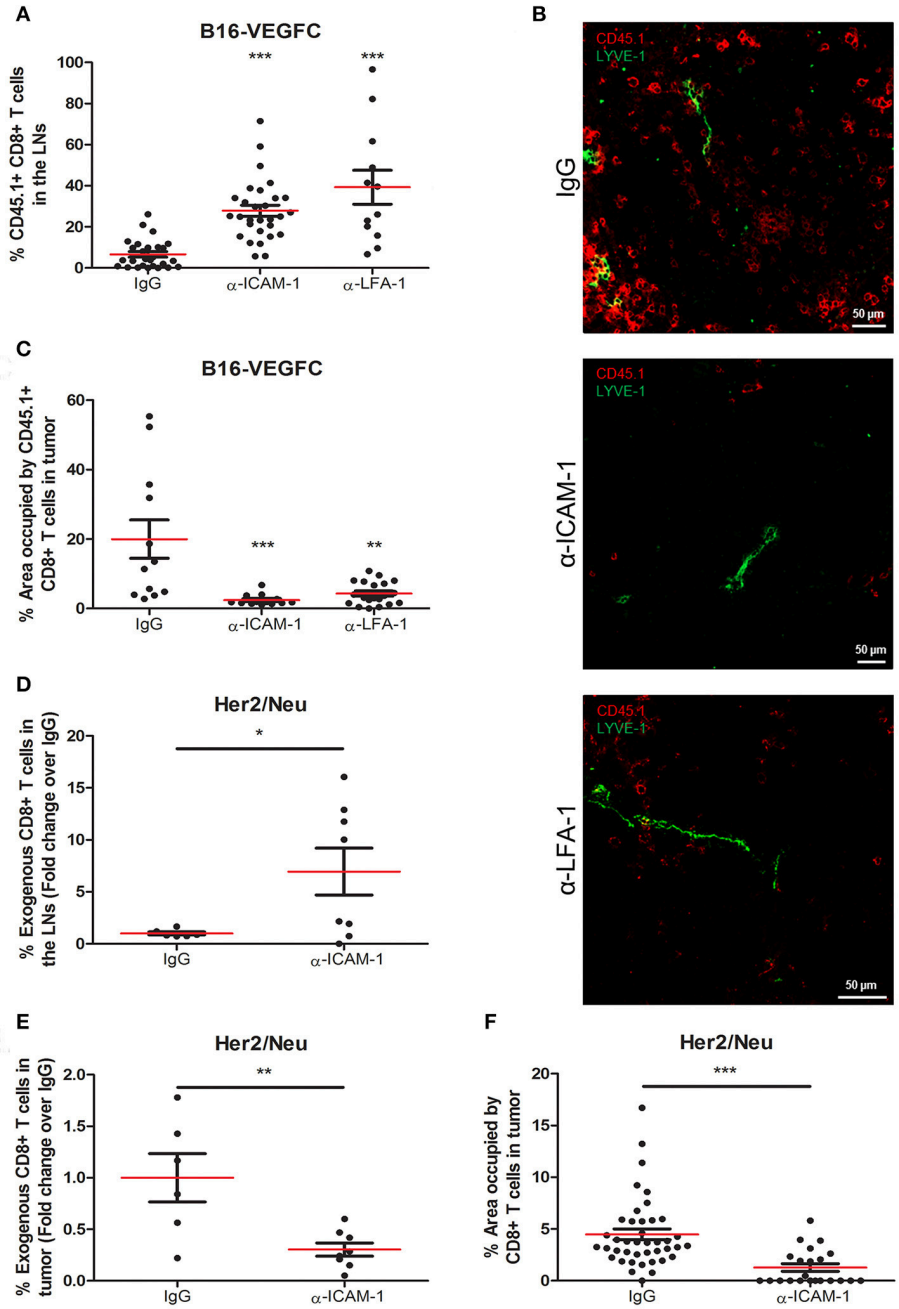
Systemic ICAM-1 blockade increases the egress of intratumorally injected CD8+ T-cells to draining LNs. **(A)** Percentages of total CD45.1+ CD8+ T cells injected in B16-VEGFC tumors that reached the draining LNs 48 h after isotype (*n* = 29), anti-ICAM-1 (*n* = 32), or anti-LFA-1 (*n* = 12) treatment and detected by flow cytometry. **(B)** Representative confocal images of B16-VEGFC tumors in mice treated with isotype, anti-ICAM-1 or anti-LFA-1 antibodies showing CD45.1+ T cells (red) and intratumoral LVs (green). **(C)** Percentages of CD45.1+ CD8+ T-lymphocytes remaining in B16-VEGFC tumors 48 h after isotype, anti-ICAM-1 or anti-LFA-1 antibody treatment. Percentage of positive area was calculated using a manual region of interest (ROI) based on CD45.1 signal. At least 12 pictures from 3 different tumors where analyzed in each case. **(D)** Percentages of total CD8+ T cells injected in Her2/Neu tumors that reached the draining LNs after isotype (*n* = 6) or anti-ICAM-1 (*n* = 8) treatment and detected by flow cytometry. **(E)** Percentages of total CD8+ T cells injected in Her2/Neu tumors that remained in tumors 48 h after isotype (*n* = 6) or anti-ICAM-1 (*n* = 8) treatment and detected by flow cytometry. **(F)** Percentages of CD8+ T-lymphocytes remaining in Her2/Neu tumors 48 h after isotype or anti-ICAM-1 antibody treatment. Percentage of positive area was calculated using a manual region of interest (ROI) based on CD8 signal. At least 23 pictures from 3 different tumors where analyzed in each case (*p < 0.05, **p < 0.01, ***p < 0.001).

Our main objective was to study whether ICAM-1 played a role in the egress of antigen experienced T cells from tumors to the lymph nodes. To study this issue, we administered intratumorally OT1 T cells that had been previously activated *in vitro* by exposition to OVA antigen. Specifically, we injected 5 × 10^5^ *in vitro*-activated OT1 T cells intratumorally into B16-VEGFC mouse melanoma tumors [with intense lymphatic vascularization (25), Supplementary Figure 1A] that had been pre-treated 8 h earlier with an ICAM-1 blocking antibody. Two days later, mice were sacrificed and tumors and lymph nodes were extracted and processed for T-cell detection by immunofluorescence and flow cytometry. Contrarily to what we expected, we observed significantly higher counts of T-lymphocytes reaching the draining lymph nodes in mice treated with an anti-ICAM-1 antibody than in isotype (IgG) treated animals (Figure 1A). LFA-1 (αLβ2) is the integrin ligand for ICAM-1. In order to prove that ICAM-1/LFA-1 interaction was responsible of T cell retention, we performed the experiments pre-blocking the β2 integrin LFA-1 instead. As occurred with ICAM-1 blockade, this strategy resulted in incremented T cell emigration from tumors toward draining lymph nodes (Figure 1A). Accordingly, we also observed that the number of adoptively transferred T cells remaining in tumors varied accordingly, being higher in control than in ICAM-1 or LFA-1 treated mice (Figures 1B,C).

We then postulated that the incremented CD8+ T cell retention was due to ICAM-1 mediated lymphocyte-tumor interaction. To test this, we first detected by flow cytometry the intensity of ICAM-1 expression on the surface of tumor cells. As shown in Supplementary Figure 1B, B16-melanoma cells express ICAM-1 on their surfaces at significantly lower (10-fold less) levels than T cells, in accordance with what had already been described (26). Moreover, when we analyzed the adhesion of T cells to monolayers of B16-VEGFC melanoma cells, we observed very weak adhesion that did not change regardless of the assays being done in the presence of anti-ICAM-1 antibodies (Supplementary Figure 1C). These results lead us to postulate that ICAM-1 blockade may contribute to diminished T cell retention in tumors by mechanisms different from direct T-cell adhesion to tumor cells.

Next, to clarify whether our observations were exclusive of the B16-VEGFC tumor model, we performed the ICAM-1 blocking experiments in Her2/Neu transgenic mice bearing spontaneous breast tumors. These tumors presented less intense basal T cell infiltrates than B16-VEGFC tumors in spite of possessing abundant intra- and peritumoral lymphatic vasculature (Supplementary Figure 1D). In this case, we were able to induce higher migration of intratumorally-injected CD8+ T-lymphocytes to draining lymph nodes in mice pre-treated with ICAM-1 blocking mAbs (Figure 1D). In the same sense, the amount of T-lymphocytes that remained in the tumor tissue was significantly decreased in anti-ICAM-1 treated mice (Figure 1E). We confirmed these results by immunofluorescence-based inspection of the Her2/Neu tumors with clear reductions in the number of CD8+ T-lymphocytes in tumors derived from ICAM-1 pre-treated animals (Figure 1F). These results confirmed that the effect observed was not model-dependent.

### ICAM-1 blockade increments the egress of *in vivo* activated T cells

**Figure 2:**
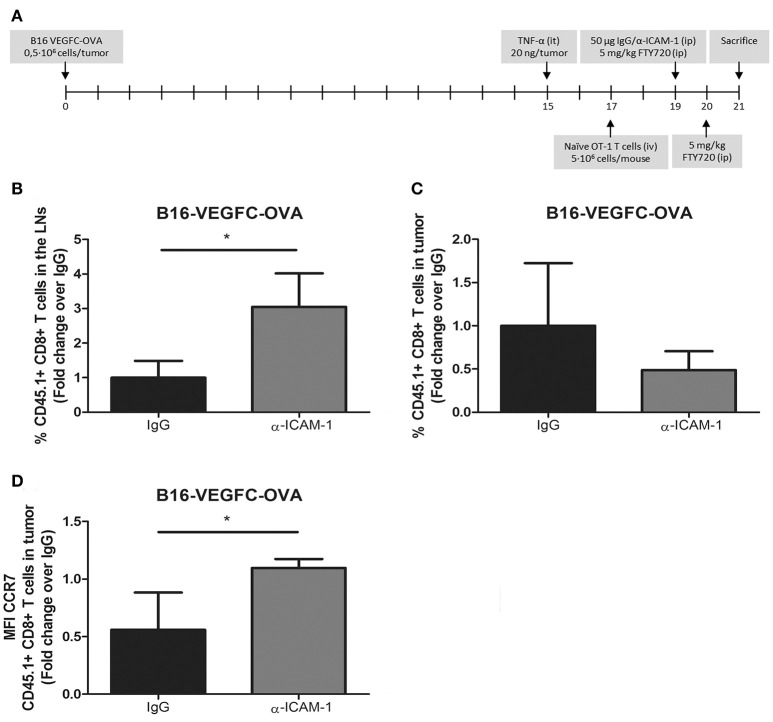
*In vivo* activated T-cells experience increased migration from tumors to the LNs after ICAM-1 blockade. **(A)** Treatment schedule. **(B)** Percentages of intravenously injected CD45.1+ CD8+ T cells in the draining LNs 48 h after treatment with isotype (*n* = 9) or anti-ICAM-1 (*n* = 6) antibodies of mice bearing B16-VEGFC-OVA tumors. Cells were detected by flow cytometry. **(C)** Percentages of intravenously injected CD45.1+ CD8+ T cells that remained in B16-VEGFC-OVA tumors 48 h after treatment with isotype (*n* = 9) or anti-ICAM-1 (*n* = 6) antibodies and detected by flow cytometry. **(D)** Mean fluorescence intensity of CCR7 expression in CD45.1+ CD8+ T cells remaining in B16-VEGFC-OVA tumors in mice treated as in C. (*p < 0.05).

Up to this moment, our experimental set up comprised the intratumoral injection of high numbers of exogenously activated OT1 T cells. Next, we wanted to test whether our results were reproducible in a more physiologic experimental context. For that purpose, we introduced naïve OT1 CD8+ T cells intravenously in mice harboring pre-inflamed subcutaneous B16-VEGFC-OVA melanomas, which present intense lymphatic vascularization and express OT-1 cognate antigen OVA (Supplementary Figure 2A), allowing them to infiltrate tumors for 48 h. At this time point, mice were treated i.p. with FTY720 (fingolimod) to block further T-cell egress from the lymph nodes and half of the mice were treated with i.p. injections of ICAM-1 blocking antibodies. Two days later, mice were sacrificed and the number CD8+ OT1 T lymphocytes in tumors and lymph nodes were quantified by flow cytometry. With this experimental setting (Figure 2A) we assumed that the differences observed in OT1 T cell counts in the lymph nodes were mostly accountable to cell egress from tumors and not to increased recirculation from the lymph nodes. In fact, OT1 cell counts in peripheral blood of fingolimod-treated animals were almost negligible 24 and 48 h after treatment with this drug (Supplementary Figure 2B). In this new *in vivo* setting, ICAM-1 blockade was also able to increment the amount of OT1 T cells present in the lymph nodes of treated animals and showed a tendency to minor (albeit not significant) incidence of OT1 T cells remaining in tumors (Figures 2B,C).

T-cell migration to the lymph nodes occurs through chemokine mediated guidance being CCL21 (27) one of the main chemokines responsible for their recruitment. We then checked the expression of the chemokine receptor CCR7 that binds to the lymphatic attracting chemokine CCL21 in tumor infiltrating OT1 T cells. As depicted in Figure 2D, we detected significant increments in the expression of CCR7 in intratumoral OT1 T cells from anti-ICAM-1 treated animals. With these results we demonstrate how transient blockade of ICAM-1 may improve intratumoral T cell migration to the lymph nodes and favor the intra tumor expression of one of the most important lymph node chemotactic receptors.

### Migration of ICAM-1 blocked T-lymphocytes is dependent on GPCRs

**Figure 3:**
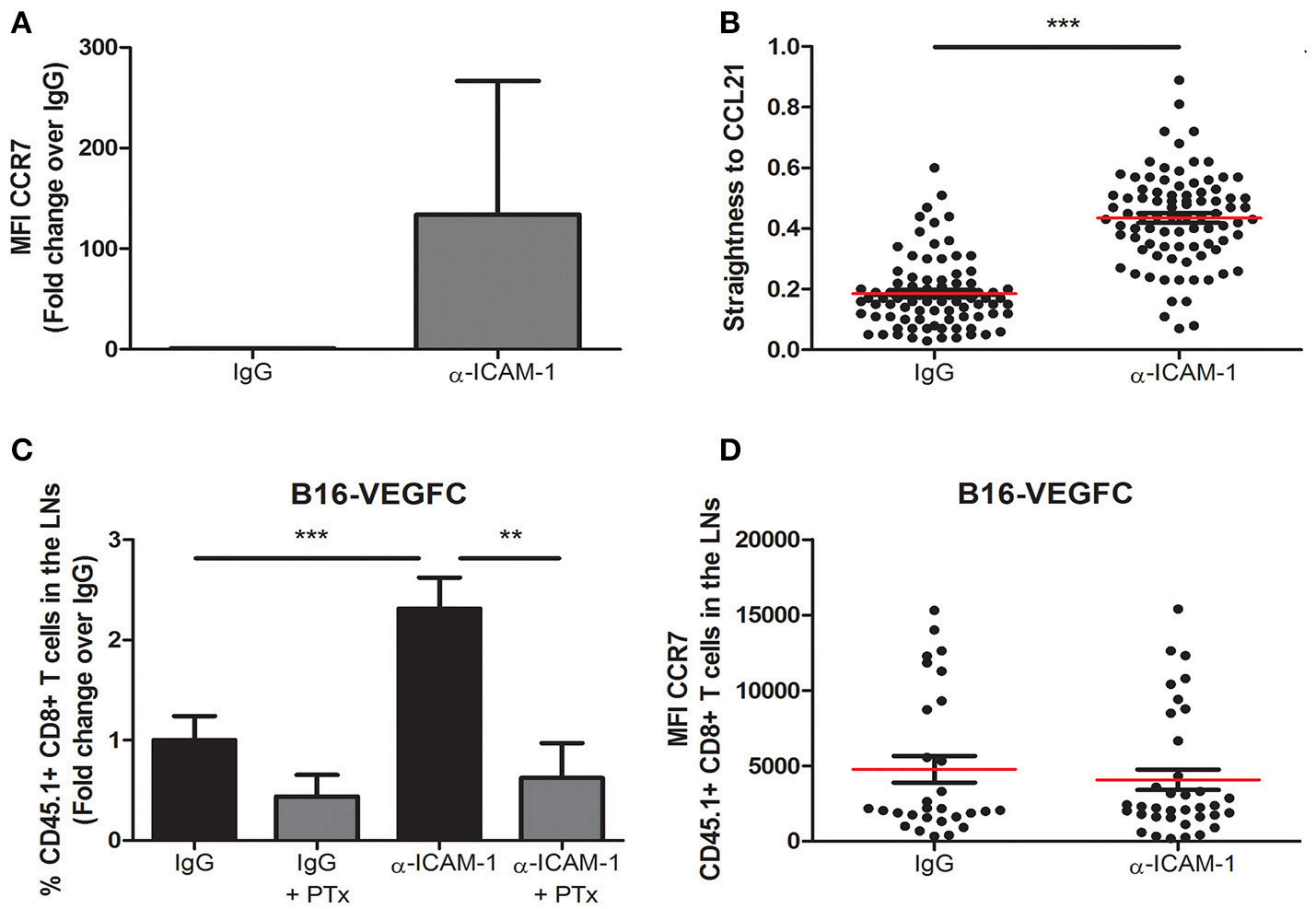
Integrin ligand blockade leads to increments in chemokine-dependent migration. **(A)** Surface expression of the chemokine receptor CCR7 on *in vitro* activated OT-1 CD8+ T cells in the presence or in the absence of anti-ICAM-1 antibodies. Flow-cytometry data was collected from three or more independent experiments. **(B)** Chemokine-directed migration of activated CD45.1+ CD8+ T lymphocytes in 3D collagen gels after being treated with anti-ICAM-1 antibodies or control IgG for 48 h. Cell movement toward CCL21 gradients was recorded by *in vivo* confocal microscopy and cell straightness was analyzed using IMARIS software. Each dot represents a single tracked cell. At least 80 cells were tracked in each case. **(C)** Percentages of intratumorally-injected CD45.1+ CD8+ T cells that reached the draining LNs 48 h after treatment with isotype antibody (*n* = 35), isotype + PTx (*n* = 6), anti-ICAM-1 antibody (*n* = 39), or anti-ICAM-1 + PTx (*n* = 10). **(D)** Mean fluorescence intensity of CCR7 expression in LN CD45.1+ CD8+ T cells 48 h after treatment with isotype (*n* = 26) or anti-ICAM-1 (*n* = 33) antibodies. Cells were detected by flow cytometry (**p < 0.01, ***p < 0.001).

Once we had sufficiently proved the reproducibility of the effect of transient ICAM-1 blockade in CD8+ T cell egress from tumors, we aimed at searching the cellular mechanism behind it. Therefore, in the first place we determined the expression of the chemokine receptor CCR7 in lymphocytes activated *in vitro* in the presence or in the absence of anti-ICAM-1 antibodies. As shown in Figure 3A, ICAM-1 blockade was again associated with increments in the expression of CCR7 on the surface of CD8+ T-lymphocytes. To find out if these changes in CCR7 expression were responsible for the improved cell migration, we measured *in vitro* cell migration toward gradients of CCL21 in ICAM-1 blocked or isotype (IgG) pre-treated OT1 cells. In these assays, we observed significant increments in cell directionality toward gradients of CCL21 (Figure 3B) in ICAM-1 pre-blocked T-lymphocytes.

To provide an *in vivo* correlate for these findings, we again injected CD8+ T-lymphocytes in B16-VEGFC tumors from mice that had been or not pre-treated with anti-ICAM-1 antibodies but, in this case, half of the mice were pre-treated with the G-protein inhibitor Pertussis Toxin 4 h before OT1 transfer. This inhibitor precludes signaling through chemokine receptors. Notably, inhibition of G-protein signaling abrogated ICAM-1 induced increments in T cell migration (Figure 3C). Moreover, when we compared the expression of CCR7 in OT-1 lymphocytes that had reached the lymph nodes from anti-ICAM-1 or IgG treated mice, we did not find differences between groups (Figure 3D). Hence, differential CCR7 expression after ICAM-1 blockade is transient and occurs in tumor stroma infiltrating lymphocytes.

Therefore, our results demonstrate that inhibition of ICAM-1 augments chemokine-signaling conducive to T cell migration to the lymph nodes.

### Transient ICAM-1 blockade diminishes the occurrence of intratumoral T cell clusters

Next, we wondered whether augmented T cell egress after ICAM blockade was due to changes in the viability of tumor infiltrating lymphocytes or alterations in lymphatic vessels phenotype after treatment. To that aim, the viability of exogenously administered CD8+ T lymphocytes was analyzed by zombie nir staining, but we did not find any increment in the amount of dead cells neither in the tumor nor in the LNs of B16-VEGFC bearing mice (Supplementary Figure 3A). When we checked PD-1 expression of intratumorally injected CD45.1 CD8+ T lymphocytes we did not find any difference either (Supplementary Figure 3B). Besides, there were also no changes in the viability of CD8+ T lymphocytes transferred to Her2/Neu tumors (Supplementary Figure 3C).

**Figure 4:**
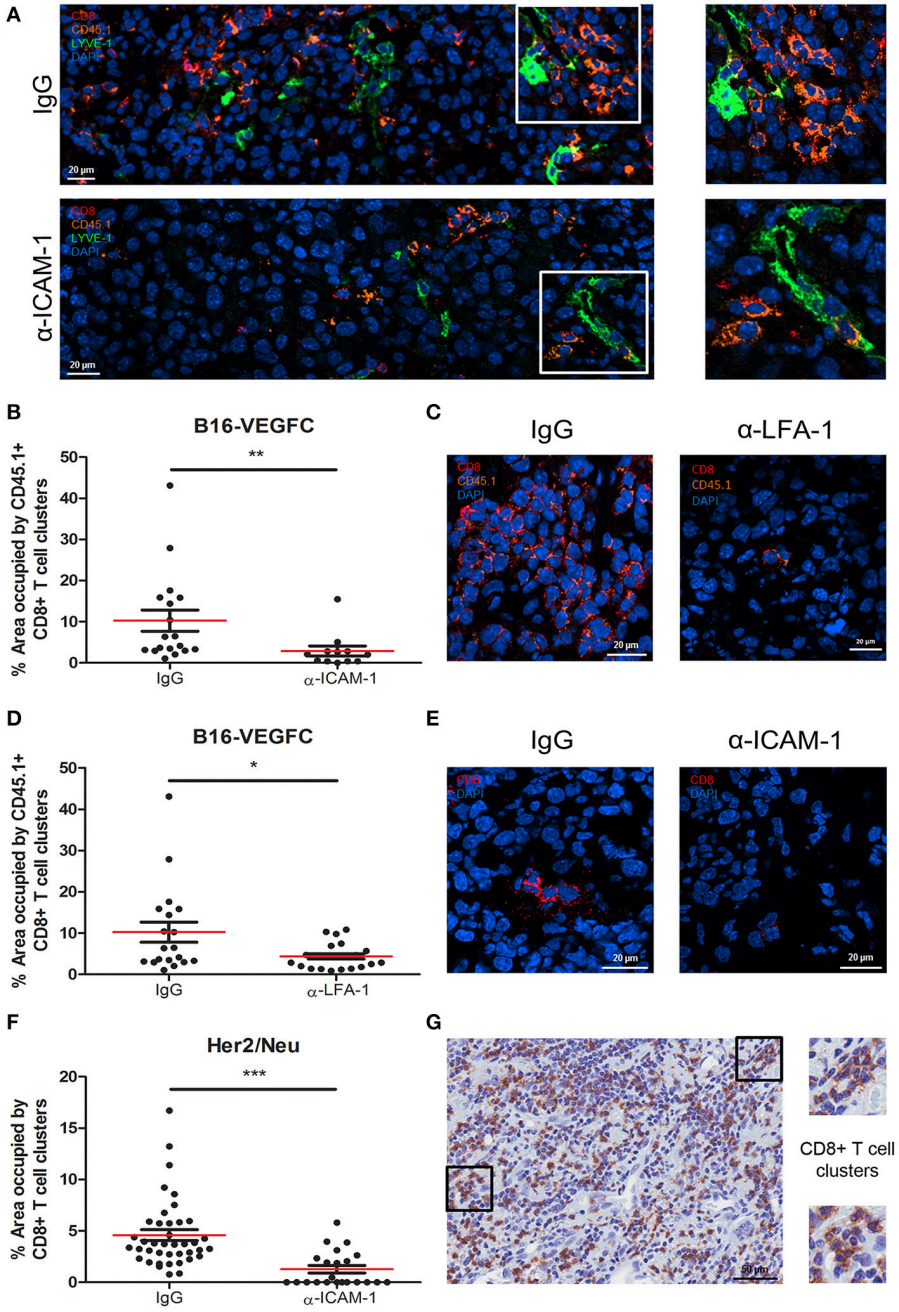
Intratumoral CD8+ T-cells form diffuse cell-clusters that are reduced upon anti-ICAM-1 treatment. **(A)** Representative confocal images of B16-VEGFC tumors from isotype or anti-ICAM-1 treated mice showing CD45.1+ T cells (orange), CD8+ T cells (red), and intratumoral LVs (green). Images at the right end show magnifications of T cell clusters. **(B)** Percentages of area occupied by CD45.1+ CD8+ T-lymphocyte clusters in B16-VEGFC tumors 48 h after isotype or anti-ICAM-1 antibody treatment. Percentage of total area occupied by T cell clusters was calculated using a manual quantification of aggregates of four or more cells. At least 13 pictures where analyzed in each case. **(C)** Representative confocal images of B16-VEGFC tumors from isotype or anti-LFA-1 treated mice showing CD45.1+ T cells (orange) and CD8+ T cells (red). **(D)** Percentages of area occupied by CD45.1+ CD8+ T-lymphocyte clusters in B16-VEGFC tumors 48 h after isotype or anti-LFA-1 antibody treatment. Percentage of total area occupied by T cell clusters was calculated using a manual quantification of aggregates of four or more cells. At least 19 pictures where analyzed in each case. **(E)** Representative confocal images of Her2/Neu tumors in isotype or anti-ICAM-1 treated mice showing CD8+ T cells (red). **(F)** Percentages of area occupied by CD8+ T-lymphocyte clusters in Her2/Neu tumors 48 h after isotype or anti-ICAM-1 antibody treatment. Percentage of total area occupied by T cell clusters was calculated using a manual quantification of aggregates of four or more cells. At least 26 pictures where analyzed in each case. **(G)** Representative microphotograph showing CD8+ T cell clusters as detected by immunohistochemistry on samples obtained from a series of 10 human melanoma patients. Magnifications show two instances of T cell clusters (*p < 0.05, **p < 0.01, ***p < 0.001).

Microscopic inspection of B16-VEGFC tumors showed no morphological changes in intra- or peri-tumoral lymphatic vessels in mice treated with ICAM-1 blocking antibodies (Supplementary Figure 3D). However, it called our attention to observe intratumoral T-cell clusters in control tumors injected with activated CD8+ T cells that were significantly decreased in anti-ICAM-1 treated mice (Figures 4A,B). We also observed the same phenomenon when LFA-1 was blocked (Figures 4C,D). T-cell clustering by LFA-1 integrin mediated homotypic binding is a hallmark of T-lymphocyte activation (28).

T cell clusters are usually associated with CD11b myeloid cells (29). To inspect whether this was the case in the tumor tissue, we searched for the presence of CD11b+ myeloid cells in T cell clusters. As shown in Supplementary Figures 4A,B, CD11b+ myeloid cells were present in the vicinity of such T cell aggregates. In spite of not observing instances of T cells rossetting around CD11b+ cells, we could detect frequent images of side to side cell association. We were unable to detect any colocalization with CD11b+ cells in anti-ICAM-1 treated mice.

To rule out the possibility of a model specific effect, we evaluated the presence of T cell clusters in spontaneous breast tumors that arise in Her2/Neu transgenic mice also following intratumoral injection of activated CD8+ T cells (Figure 4E). Although at a much lower extent than in B16-VEGFC tumors, in this model we also observed the formation of T cell clusters. Importantly, we were unable to detect any T cell clusters in spontaneous tumors from ICAM-1 pre-treated animals (Figures 4E,F). Therefore, exposure to anti-ICAM-1 impedes the formation of leukocyte clusters inside the tumor tissue.

Finally, we investigated whether T cell clusters actually exist in pathological tissue specimens obtained from melanoma patients. Immunohistochemistry studies of tissue sections revealed that CD8+ T cell clusters are formed in human tumors and therefore tumors may retain T-lymphocytes through homotypic adhesive mechanisms, potentially susceptible of *in situ* ICAM-1 blockade to enhance T cell egress to the lymph nodes (Figure 4G).

### ICAM-1 blockade favors T cell de-clustering and increments migration across monolayers of lymphatic endothelial cells

**Figure 5:**
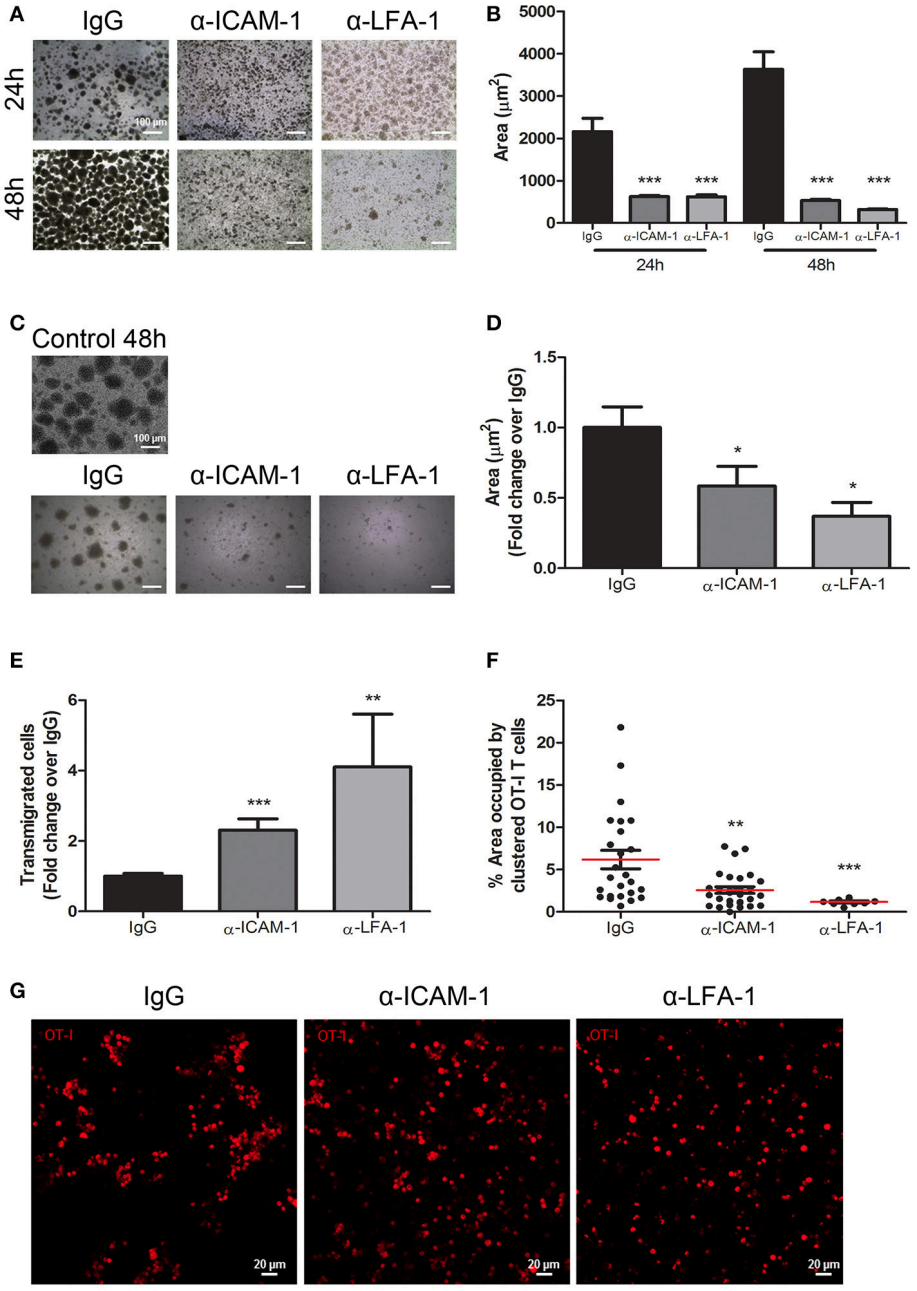
*In vitro* blockade of the ICAM-1/LFA-1 pair abolishes T cell clustering and speeds their transmigration across lymphatic monolayers. **(A)** Representative microphotographs of CD45.1+ CD8+ T cell clusters after *in vitro* activation for 24 or 48 h in the presence of control isotype or anti-ICAM-1 or anti-LFA-1 antibodies. **(B)** Quantification of T cell cluster formation in A calculated as the percentage of total area that is occupied by T-cell clusters. Pooled data from at least two independent experiments are shown. **(C)** Representative microphotographs showing destruction of already-formed T cell clusters of CD45.1+ CD8+ activated T lymphocytes being subsequently treated for 24 h with isotype control, anti-ICAM-1 or anti-LFA-1 antibodies. **(D)** Quantification of T cell clusters was performed as in **B**. **(E)** Transmigration of activated CD45.1+ CD8+ T cells for 16 h across monolayers of IMLEC through serum-containing medium. The assays were performed in the presence of anti-ICAM-1 or anti-LFA-1 antibodies or control IgG. Data from five independent experiments are shown. **(F)** Percentages of CD8+ T-lymphocytes remaining in the upper well of Boyden chambers after transmigration assays as in E. Percentages of total area occupied by T-cell clusters was calculated using a manual region of interest (ROI) based on CD8+ signal. At least nine pictures where analyzed in each case. **(G)** Representative confocal images of OT1 CD8+ lymphocytes (red) that remained in the upper well of transwell Boyden chambers after transmigration experiments as in E (*p < 0.05, **p < 0.01, ***p < 0.001).

To further support the importance of ICAM-1-mediated T-cell aggregation to impede migration, we performed *in vitro* CD8+ T cell clustering and de-clustering assays and subsequently checked lymphocyte migration. First, we performed *in vitro* CD8+ T-lymphocyte clustering assays in the presence of anti-ICAM-1 or anti-LFA-1 antibodies following activation with an OVA-derived peptide. ICAM-1 and LFA-1 blockade significantly diminished T-cell aggregation (Figures 5A,B). When we checked by flow cytometry the proliferation (CSFE dilution), activation (CD25 staining), viability (7-AAD staining), and apoptosis (caspase 3) we could not find any significant difference between groups (Supplementary Figure 5), therefore ICAM-1 pre-treatment does not affect activation. In parallel experiments, we studied whether the presence of ICAM-1 or LFA-1 blocking antibodies were able to disaggregate the already formed T cell clusters. Reversion of the aggregates took place as shown in Figures 5C,D. We then evaluated the transmigration of clustered CD8+ T cells across monolayers of lymphatic endothelial cells in the presence or in the absence of ICAM-1/LFA-1 blocking antibodies and verified significant increments in transmigration when the assays were performed in the presence of anti-ICAM-1 or anti-LFA-1 antibodies (Figure 5E). Not surprisingly, when we checked the upper well of the Boyden chambers by confocal microscopy we discovered large CD8+ T cell clusters in control isotype treated cells (Figures 5F,G). From these results we suggest that one mechanism behind ICAM-1 blockade incremented out-migration from tumors might be disaggregating T cell clusters, and thus facilitating single cell migration across the lymphatic endothelium into the vessel lumen.

To sum up, transient ICAM-1 blockade significantly affects the migratory properties of intratumoral leukocytes.

## Discussion

In this study we experimentally support a mechanism of T-cell retention in tumors by LFA-1/ICAM-1 mediated leukocyte aggregation. Such a mechanism seems to prevent CD8+ T cell recirculation. The presence of T cells forming aggregates in human tumors has been broadly described and their composition vary from simple clusters of lymphocytes to more complex structures reminiscent of secondary lymphoid organs (30, 31).

In the present work, we demonstrate the presence of small diffuse aggregates of T lymphocytes in human melanoma tissue section and in animal models of melanoma and breast carcinoma. In our experimental models, blockade of ICAM-1 before intratumoral delivery of activated CD8+ T cells resulted in decrements in intratumoral T-cell clusters and in the amount of intratumoral CD8+ T-cells. Importantly, these results were accompanied by augmented CD8+ T cell arrival to draining lymph nodes. Blockade of LFA-1 in parallel experiments produced the same results as when animals were treated with anti-ICAM-1 antibodies, proving that ICAM-1/LFA-1 axis mediates the modulation of lymphocyte egress from tumors. Furthermore, we observed the same effect when ICAM-1 was blocked 48 h after intravenous injection of naïve T-cells recognizing tumor antigens.

T cell clustering or aggregation has been considered a hallmark of *in vitro* and *in vivo* T-cell activation (32). In this process, LFA-1/ICAM-1 adhesion facilitates T-cell activation through their contribution to the immunological synapse (33). As a result, T cell proliferation and differentiation are orchestrated through the activation of signaling cascade events. For example, adhesive contacts between T-cells and antigen presenting cells serve to exchange cytokines which are important for T cell activation, such as IFNγ and IL-2 (29, 34, 35). Although it was established that T cells deficient in ICAM-1 exhibit phenotypes of terminally differentiated cells (28), in our hands *in vivo* short-term ICAM-1 blockade did not alter the expression of activation markers such as the immune checkpoint inhibitor PD-1 neither produced any difference in the viability of tumor infiltrating leukocytes. In this context, it is interesting to bring in a recent work published by R. Alon and co-workers in which the authors demonstrate how ICAM-1/LFA-1 interactions are dispensable for functional immune synapses between dendritic cells and CD4+ T cell in the lymph nodes (36).

In our view, de-construction of ICAM-1 mediated T cell clusters leads to the release of single T cells to freely migrate and enter the lymphatic vasculature. In fact, tumors are not the only instance of ICAM-1-mediated T-cell retention. For example, antigen-dependent intrahepatic CD8+ T cells are restrained from emigration by LFA-1/ICAM-1 mediated mechanisms (37). In this vein, LFA-1 is used by liver-resident T cells as a molecular switching device to patrol the hepatic sinusoids being their engagement dependent on stromal expression of ICAM-1 (38).

In search for a mechanism that may contribute to the increased migration observed in anti-ICAM-1 treated animals, we looked for differences in the expression of chemokine receptors by intratumoral T cells after ICAM-1 blockade and observed higher expression of the CCL21 receptor CCR7. Increased expression of this chemokine receptor was consistent with greater *in vitro* directional migration toward cognate chemotactic cues. In this framework, it is known that lymphatic endothelial cells produce CCL21 and how dendritic cell adhesion to lymphatic vessels induces the release of CCL21 depots by lymphatic endothelial cells that contribute to attract and increment their transmigration (39). The mechanism of CCR7 upregulation after ICAM-1 blockade remains to be elucidated. In accordance to the role of CCR7, *in vivo* treatment with pertussis toxin returned T cell migration to the LNs to baseline in ICAM-1 blocked animals. Interestingly, it has been described that T cell migration through the interstitium occurs independently of integrin-mediated interactions. In this situation, T-lymphocytes spread rapidly and extend cytoplasm protrusions on stroma-immobilized CCL21 (40). Our data supports this point because ICAM-1 blockade did not impair lymphocyte access to the lymphatic vessels but on the contrary, it was incremented. Therefore, our interpretation is that ICAM-1 blockade results in destruction of T-cell clusters and increased T cell sensitivity to CCL21 chemokine-guided migration toward the lymphatic vessels, thus facilitating their transit to the lymph nodes.

Recently, an *in vivo*-photolabeling based study described that T lymphocyte egress from tumors is diverse and highly regulated (13). In previous reports from our group and others it has been described how ICAM-1 participates in leukocyte adhesion and transmigration across the lymphatic vasculature (22, 41). In fact, although in steady-state conditions lymphocytes cross the endothelial layer through an integrin indolent process (42), under inflammation or shear stress this process switches to integrin-dependent modes (21, 43, 44). Moreover, this same integrin ligand has been involved in intravascular lymphocyte crawling inside initial lymphatic capillaries (22). In our experimental model, since systemic ICAM-1 blockade did not prevent the access of tumor egressing CD8+ T lymphocytes to the lymphatic vessels, we assume that their transit is integrin independent and would probably occur through small portals that due to small size would not fit cell aggregates of more than 2-3 T-cells (42).

**Figure 6:**
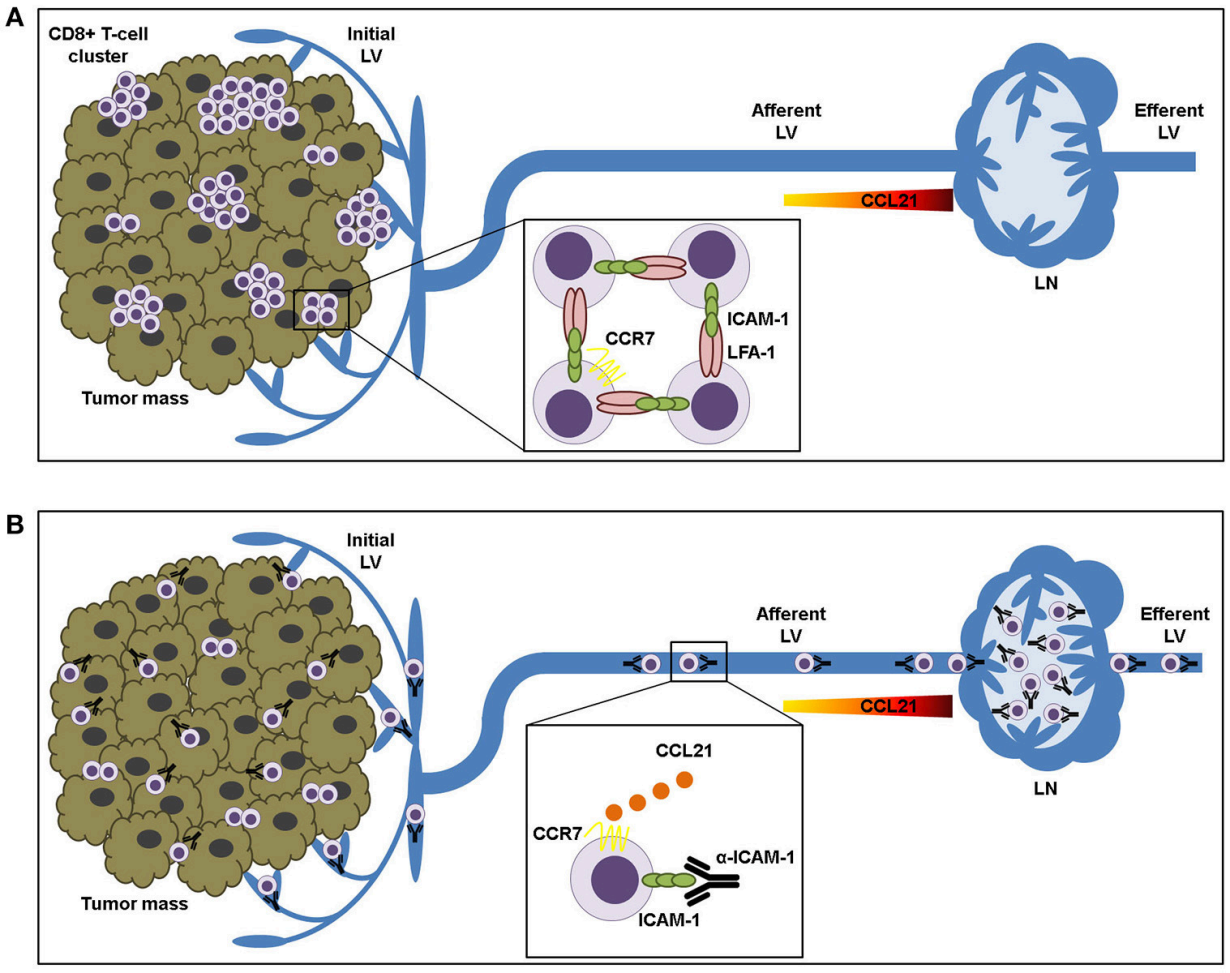
ICAM-1-LFA-1 dependent CD8+ T-lymphocyte aggregation in tumor tissue prevents recirculation to draining lymph nodes. **(A)** Intra-tumoral CD8+ T lymphocytes form clusters that are retained in the tumor microenvironment by homotypic ICAM-1-LFA-1 interactions. **(B)** ICAM-1 blockade results in destruction of T-cell clusters and increased T cell sensitivity to CCL21 chemokine-guided migration toward the lymphatic vessels, thus facilitating their transit to the draining lymph nodes.

In summary, we propose that LFA-1/ICAM-1 mechanism is not needed for T-lymphocyte transendothelial transit neither for their advancement through the tumor stroma. By contrast, it is used by intra-tumoral T cells to build up cell clusters that are retained in the tumor microenvironment (Figure 6).

Our finding that blockade of ICAM-1 increments lymphocyte migration to the lymph nodes opens important perspectives for the treatment of cancer. In this sense, we envision a treatment scenario in which ICAM-1 acts locally in combination with adoptive T-cell therapy to increment the migration of lymphocytes to the lymph nodes to establish a memory population or to expand the immune response. In our view and based on scientific grounds, the use of systemic anti-ICAM-1 antibodies as a single agent may offer important limitations. First, it is important to bear in mind that long term interference with LFA-1/ICAM-1 interactions would disrupt critical immune synapses and the entrance of leukocytes into the tumor from the bloodstream (29). In fact, there are evidences of incremented autoimmunity in mice with type 1 Diabetes Mellitus treated with anti-ICAM-1 antibodies in combination with IL-2 (45). Even more, some tumors cells express ICAM-1 on their surface or start to express it after irradiation (46). In these cases ICAM-1 is also an important target to induce a specific T-cell response against tumors. This is in the basis of using ICAM-1 sequences in the formulation of TRICOM vaccines to treat prostate cancer (47). Moreover, CAR-T cells targeting ICAM-1 are able to eliminate advanced human thyroid tumors, which express increased ICAM-1 (48). Therefore, the type of cancer to be treated with ICAM-1 blocking mAbs should be carefully selected based on its expression of ICAM-1. In our case, B16 mouse melanoma cells expressed significantly dimmer ICAM-1 than leukocytes or endothelial cells, even under inflammation.

Two recent clinical assays using BI-505, a fully human IgG1 monoclonal antibody directed against intercellular adhesion molecule-1, have been reported. The first one entailed a Phase I dose-escalation study in patients suffering from advance relapsed/refractory multiple myeloma (49). The second assay consisted in an open arm-phase 2 clinical trial enrolling four patients diagnosed with smoldering multiple myeloma and showed no relevant clinical activity although treatment was well tolerated (50). Interestingly, a Phase I/II study of BI-505 in conjunction with autologous stem cell transplant in multiple myeloma (NCT02756728) was terminated due to and adverse cardiopulmonary event in the clinical study (as reported in the informative press note released by BioInvent in November 2016). Currently, there are no evidences of any open assay using BI-505 or other anti-ICAM-1 mAb as single agent or in combination to treat cancer.

In light of the abovementioned data, we assume that anti-ICAM-1 mAbs may be more useful locally and in combination therapies. Since immunotherapy activates the immune system, and ICAM-1 blockade diminishes tumor retention and improves lymphocyte recirculation, patients with metastasis, multiple or recurrent lesions could benefit from this combination. In our view, transient treatment with ICAM-1 blocking antibodies together with immunotherapy would release cytotoxic T lymphocytes caught up in clusters, setting them free to kill tumor cells or egress to the dLNs and to subsequent lesions. In this sense, more experiments are warranted to uncover the nature of the lymphocyte subpopulations exiting the tumor and their effect in the generation of memory responses. Timing, spatial location and dosage of treatments with anti-ICAM-1 antibodies deserve further research.

## Author contributions

AY, IM, and AR conceived the study. AY and AR wrote the manuscript. AY and SG conducted the experiments. CA obtained the clinical samples and processed them. AT performed image processing and help with the statistical analysis. All authors analyzed the results and reviewed the manuscript.

### Conflict of interest statement

IM has participated in advisory boards serving Roche-Genentech, Bristol-Myers Squibb, Incyte, Medimmune, Alligator, Genmab, F-Star, Bioncotech, and Molecular Partners. IM receives grants from Bristol-Myers, Roche, and Alligator.

The remaining authors declare that the research was conducted in the absence of any commercial or financial relationships that could be construed as a potential conflict of interest.

The reviewer, CA declared a shared affiliation, with no collaboration, with several of the authors, AY, CA, IM, and AR to the handling Editor.

## Abbreviations

LFA-1: lymphocyte function-associated antigen 1
ICAM-1: intercellular adhesion molecule 1
LNs: lymph nodes
LV: lymphatic vessels
IMLEC: immortalized mouse lymphatic endothelial cells
mAb: monoclonal antibody.
