# Supplementary material for: ICAM-1-LFA-1 Dependent CD8+ T-Lymphocyte Aggregation in Tumor Tissue Prevents Recirculation to Draining Lymph Nodes
Source: Front Immunol. 2018 Sep 12;9:2084. doi: 10.3389/fimmu.2018.02084 (PMC6143661; doi:10.3389/fimmu.2018.02084)
Supplement: Supplementary file 1 [file Presentation_1.pptx]

## Slide 1
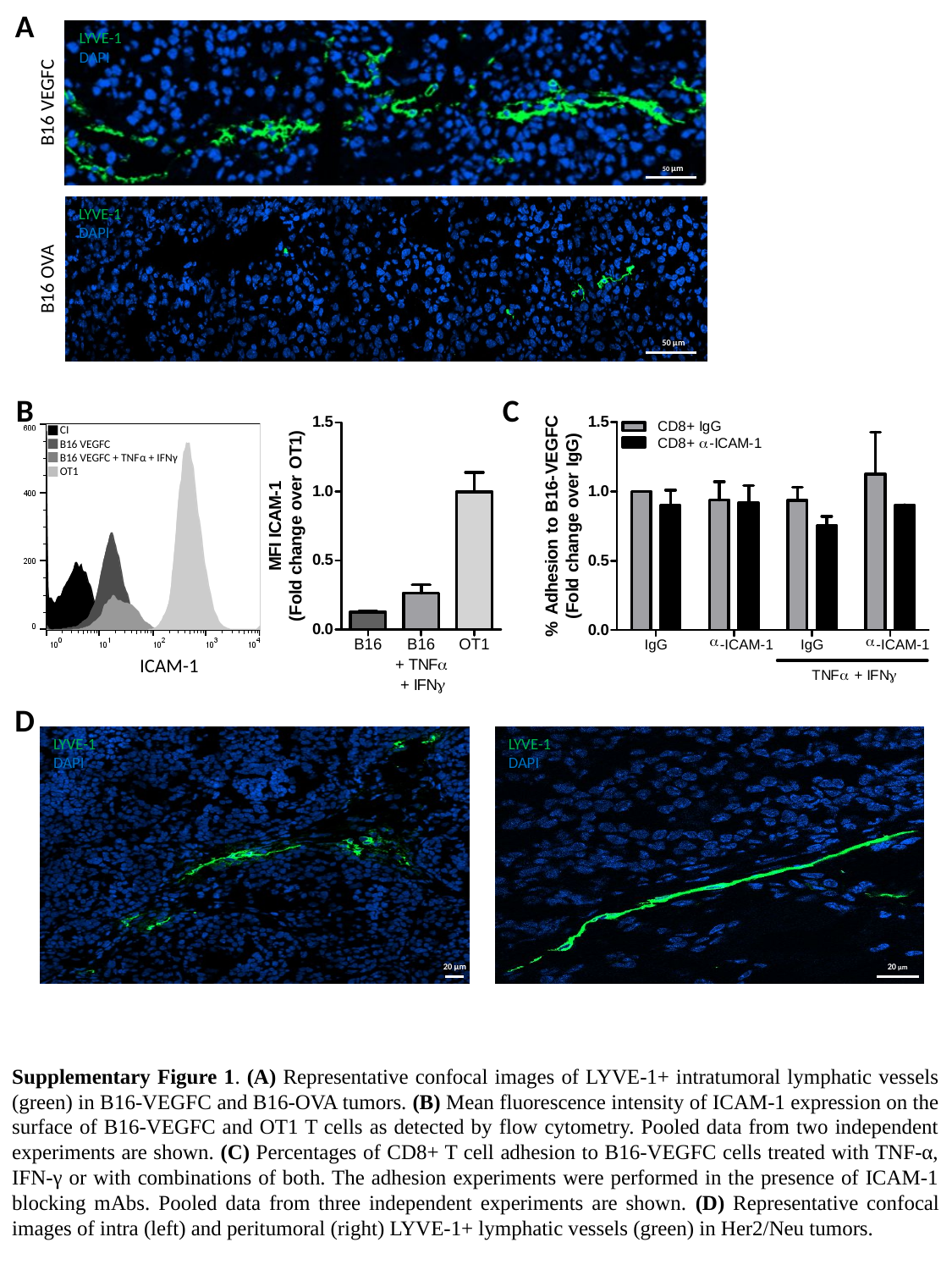

A
LYVE-1
DAPI
B16 VEGFC
50 µm
B16 OVA
50 µm
LYVE-1
DAPI
B
C
CI
B16 VEGFC
B16 VEGFC + TNFα + IFNγ
OT1
ICAM-1
D
20 µm
LYVE-1
DAPI
20 µm
LYVE-1
DAPI
Supplementary Figure 1. (A) Representative confocal images of LYVE-1+ intratumoral lymphatic vessels (green) in B16-VEGFC and B16-OVA tumors. (B) Mean fluorescence intensity of ICAM-1 expression on the surface of B16-VEGFC and OT1 T cells as detected by flow cytometry. Pooled data from two independent experiments are shown. (C) Percentages of CD8+ T cell adhesion to B16-VEGFC cells treated with TNF-α, IFN-γ or with combinations of both. The adhesion experiments were performed in the presence of ICAM-1 blocking mAbs. Pooled data from three independent experiments are shown. (D) Representative confocal images of intra (left) and peritumoral (right) LYVE-1+ lymphatic vessels (green) in Her2/Neu tumors.

## Slide 2
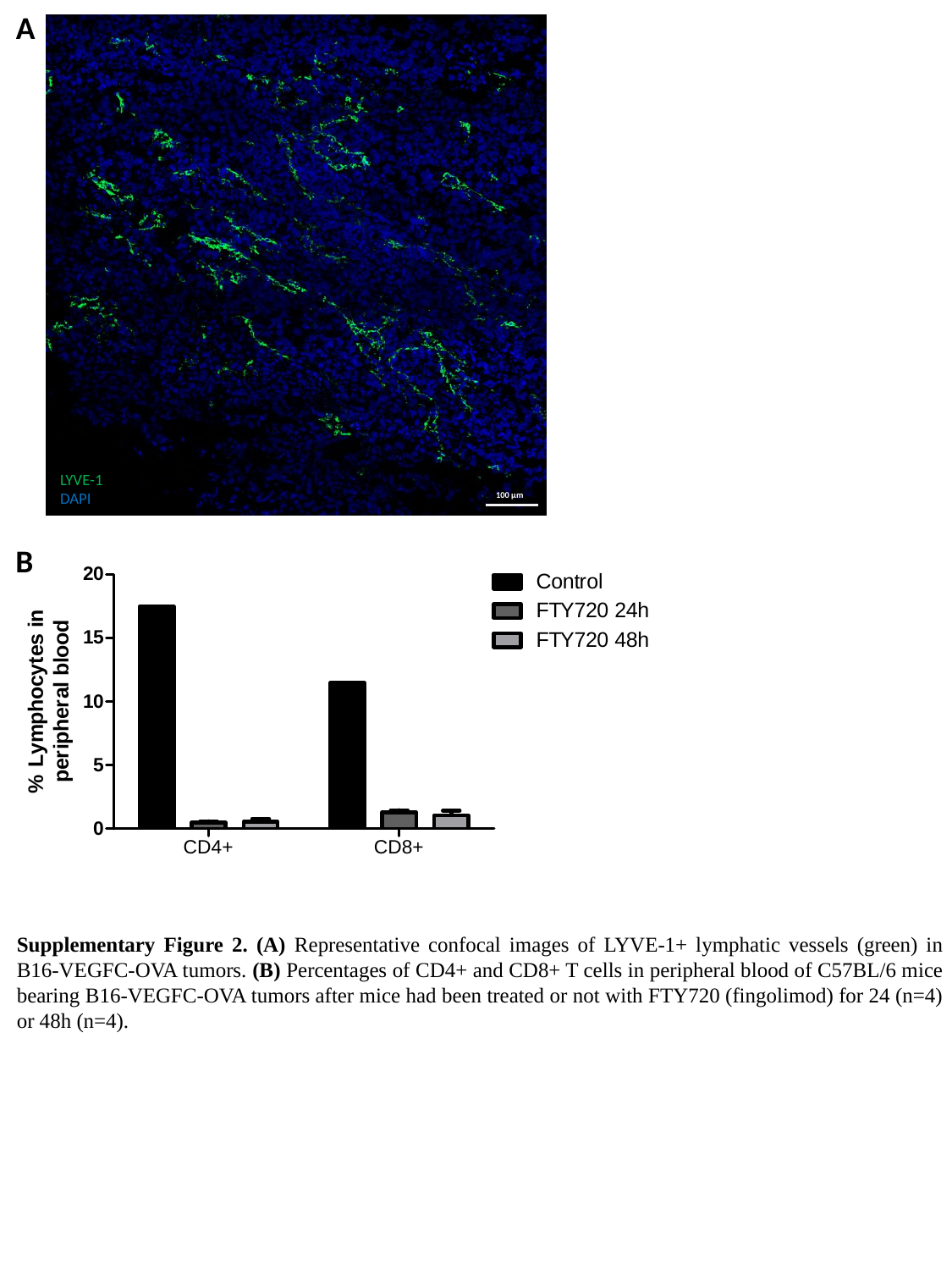

A
LYVE-1
DAPI
100 µm
B
Supplementary Figure 2. (A) Representative confocal images of LYVE-1+ lymphatic vessels (green) in B16-VEGFC-OVA tumors. (B) Percentages of CD4+ and CD8+ T cells in peripheral blood of C57BL/6 mice bearing B16-VEGFC-OVA tumors after mice had been treated or not with FTY720 (fingolimod) for 24 (n=4) or 48h (n=4).

## Slide 3
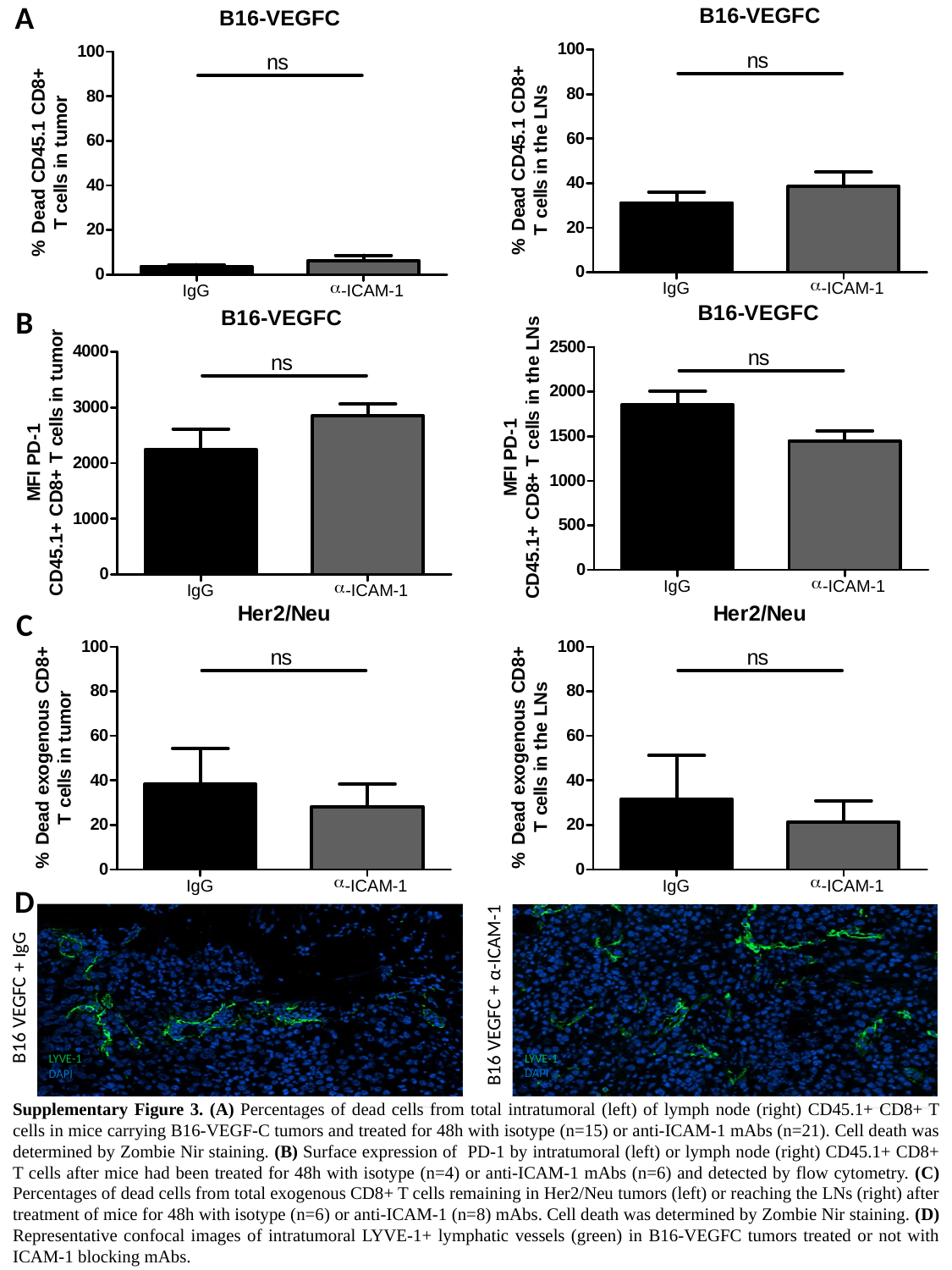

A
B
C
D
B16 VEGFC + α-ICAM-1
B16 VEGFC + IgG
LYVE-1
DAPI
LYVE-1
DAPI
50 µm
50 µm
Supplementary Figure 3. (A) Percentages of dead cells from total intratumoral (left) of lymph node (right) CD45.1+ CD8+ T cells in mice carrying B16-VEGF-C tumors and treated for 48h with isotype (n=15) or anti-ICAM-1 mAbs (n=21). Cell death was determined by Zombie Nir staining. (B) Surface expression of PD-1 by intratumoral (left) or lymph node (right) CD45.1+ CD8+ T cells after mice had been treated for 48h with isotype (n=4) or anti-ICAM-1 mAbs (n=6) and detected by flow cytometry. (C) Percentages of dead cells from total exogenous CD8+ T cells remaining in Her2/Neu tumors (left) or reaching the LNs (right) after treatment of mice for 48h with isotype (n=6) or anti-ICAM-1 (n=8) mAbs. Cell death was determined by Zombie Nir staining. (D) Representative confocal images of intratumoral LYVE-1+ lymphatic vessels (green) in B16-VEGFC tumors treated or not with ICAM-1 blocking mAbs.

## Slide 4
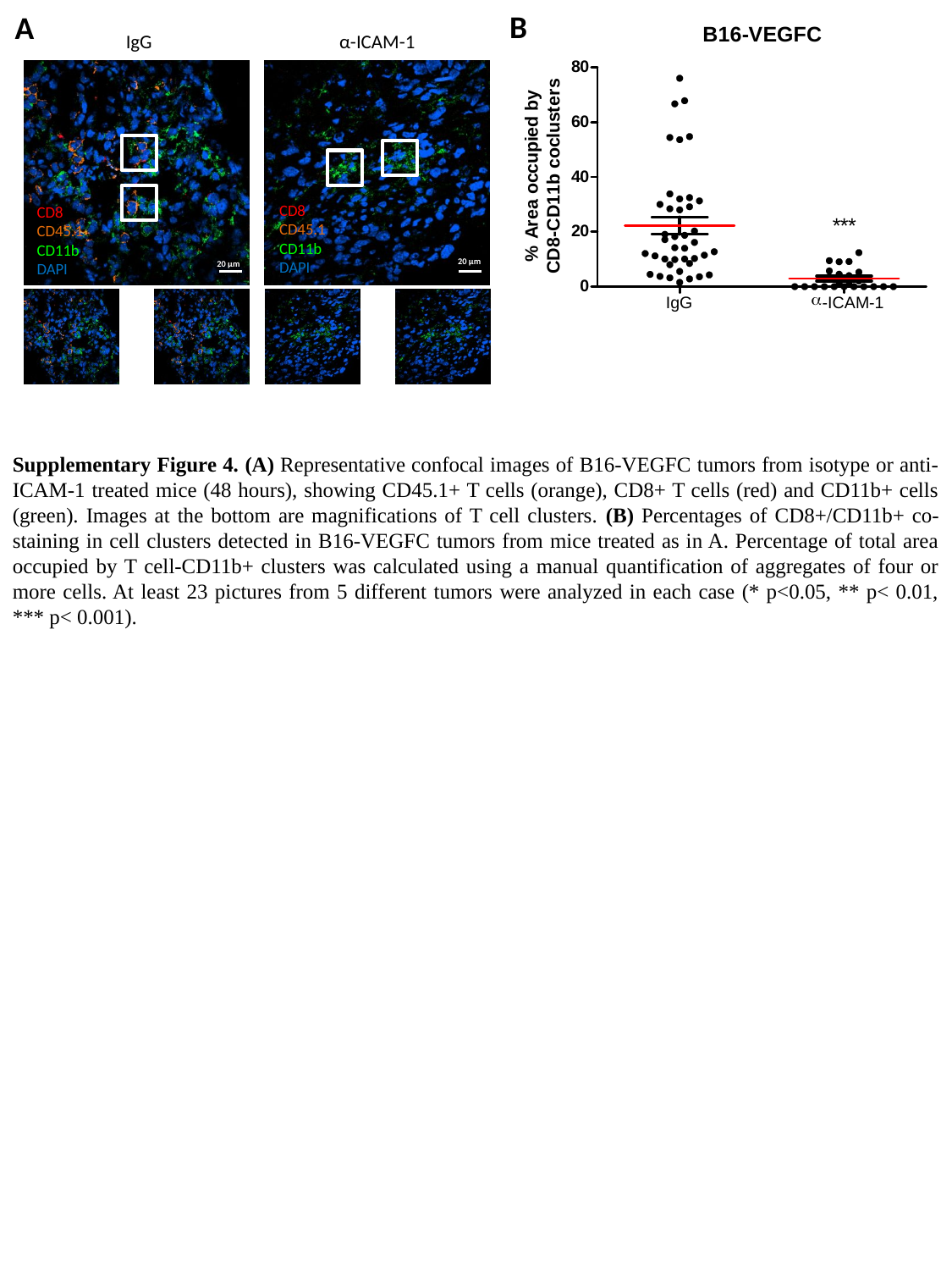

B
A
IgG
α-ICAM-1
CD8
CD45.1
CD11b
DAPI
CD8
CD45.1
CD11b
DAPI
20 µm
20 µm
Supplementary Figure 4. (A) Representative confocal images of B16-VEGFC tumors from isotype or anti-ICAM-1 treated mice (48 hours), showing CD45.1+ T cells (orange), CD8+ T cells (red) and CD11b+ cells (green). Images at the bottom are magnifications of T cell clusters. (B) Percentages of CD8+/CD11b+ co-staining in cell clusters detected in B16-VEGFC tumors from mice treated as in A. Percentage of total area occupied by T cell-CD11b+ clusters was calculated using a manual quantification of aggregates of four or more cells. At least 23 pictures from 5 different tumors were analyzed in each case (* p<0.05, ** p< 0.01, *** p< 0.001).

## Slide 5
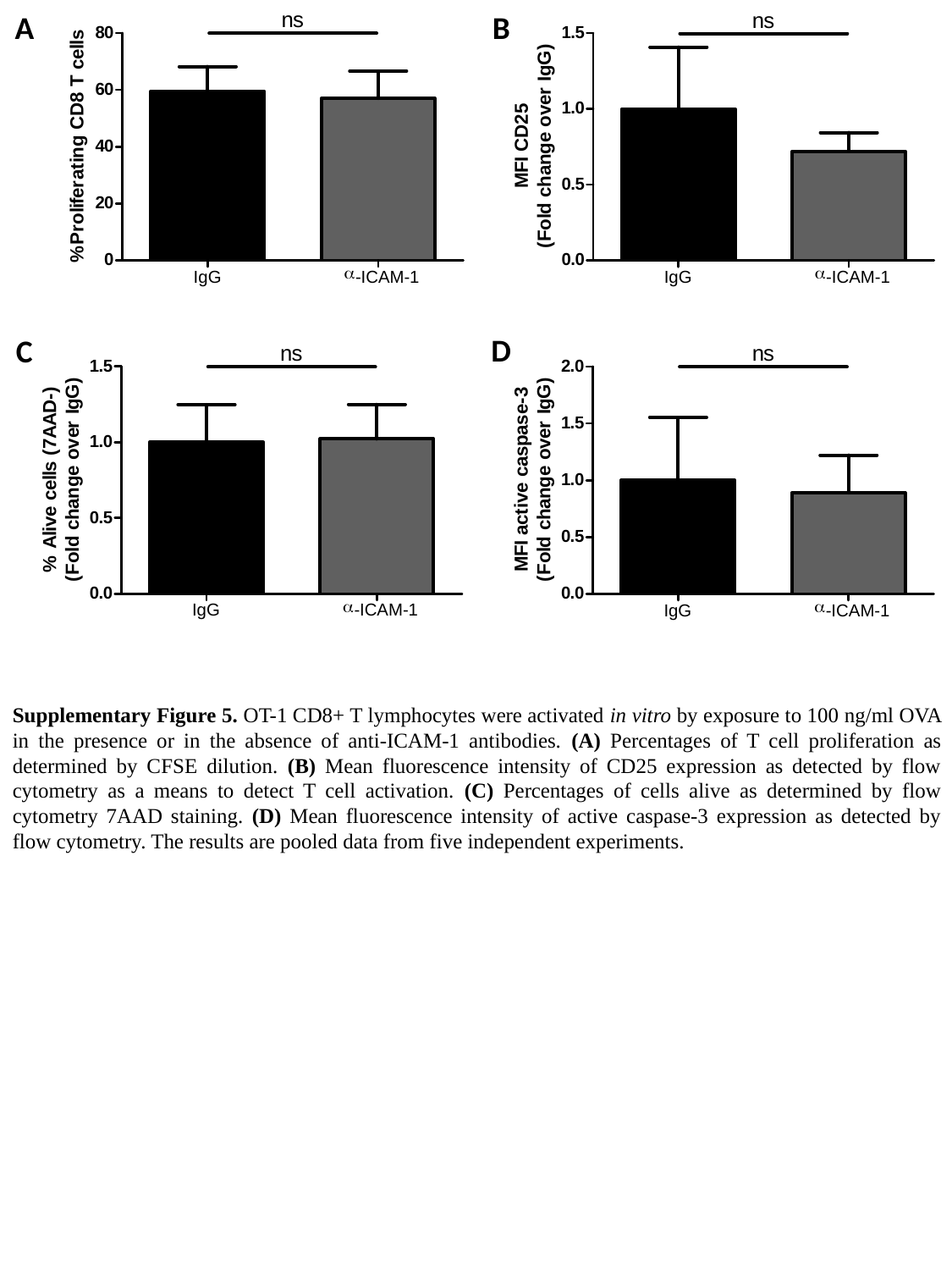

A
B
D
C
Supplementary Figure 5. OT-1 CD8+ T lymphocytes were activated in vitro by exposure to 100 ng/ml OVA in the presence or in the absence of anti-ICAM-1 antibodies. (A) Percentages of T cell proliferation as determined by CFSE dilution. (B) Mean fluorescence intensity of CD25 expression as detected by flow cytometry as a means to detect T cell activation. (C) Percentages of cells alive as determined by flow cytometry 7AAD staining. (D) Mean fluorescence intensity of active caspase-3 expression as detected by flow cytometry. The results are pooled data from five independent experiments.
